# Friends with benefits: What constitutes a ‘benefit’ in Access and Benefit Sharing (ABS) for plant genetic resources?

**DOI:** 10.1007/s10460-026-10849-x

**Published:** 2026-02-03

**Authors:** Koen Beumer, Anniek Roskam

**Affiliations:** 1 Copernicus Institute of Sustainable Development, Utrecht University, Utrecht, the Netherlands; 2 CropXR Institute, Utrecht, the Netherlands; 3 2BHonest, Laren, the Netherlands

**Keywords:** Access and benefit sharing, ABS, Nagoya protocol, Nagoya, Plant genetic resources, Just transitions

## Abstract

International conventions play an important role in regulating access to plant genetic resources. These regulations must balance the goal of ensuring wide access to plant genetic resources with doing this in a fair way. This is a central dilemma in achieving just transitions: how to move towards more sustainable societies in ways that are equitable and fair way. In regulations for plant genetic resources, this balance is struck with the concept of Access and Benefit Sharing (ABS). Under the bilateral system of the Nagoya Protocol, ABS requires anyone seeking access to a genetic resource to agree with the provider of that resource on what benefits will be shared. However, the lack of clarity as to what constitutes a ‘benefit’ has been a major stumbling block in the establishment of ABS agreements and therefore the exchange of genetic material. This article fills this gap by identifying and characterizing what types of benefits have been included in the ABS agreements established successfully under the Nagoya Protocol. We found that ABS agreements can include an incredible variety of types of monetary and non-monetary benefits, that can contribute to a wide range of different objectives, and which are not necessarily related to the benefits obtained from using the genetic resource. By providing more clarity over what benefits can be shared, this overview and characterization of benefits shared in successful ABS agreements supports the development of successful future ABS agreements. We argue that the experiences of developing Access and Benefit Sharing into a workable concept may offer valuable insights for how regulations can play a role in just transitions.

## Introduction

International conventions play an important role in regulating access to plant genetic resources. Plant genetic resources refers to “the diversity of genetic material contained in traditional varieties and modern cultivars grown by farmers as well as crop wild relatives and other wild plant species” (FAO 1997). These resources are the main ingredients for plant breeders to develop resilient cultivars that can withstand the wide range of abiotic and biotic stresses associated with climate change. As such, ensuring access to these resources is crucial in ensuring food security and tackling sustainable development challenges.

One of the main challenges these regulations face is to balance ensuring wide access to plant genetic resources with doing so in a fair way. In the case of innovative modern cultivars, this balance is struck through intellectual property rights that offer temporary monopolies to actors who have devoted time and effort to develop new cultivars. For traditional varieties and crop wild relatives, however, such regulations were long absent. While this provided unrestricted access, it was perceived to be detrimental to fairness. Actors could freely take plant genetic resources from traditional varieties, use these to develop new cultivars, and privately appropriate these via intellectual property rights without sharing the benefits with local communities who developed and conserved these genetic resources in the first place – a phenomenon also known as biopiracy (Hamilton 2008; Mbgeoji 2014). In response to this issue, several regulatory frameworks have emerged, including the International Treaty for Plant Genetic Resources for Food and Agriculture (henceforth ITPGRFA or ‘International Treaty’) that applies to a limited list of 64 crops that are crucial for food security, and the Nagoya Protocol on Access and Benefit Sharing (henceforth ‘Nagoya’) that applies to all other genetic resources.

A key concept by which these regulations aim to strike the balance between access and fairness is the concept of Access and Benefit Sharing (ABS). This concept stipulates that actors who want to access plant genetic resources (‘users’), must share some of the benefits that result from their use with the providers of those resources (CBD 2011b). The underlying idea is that ABS provides an incentive for ‘providers’ to conserve and share plant genetic resources in return for getting a share of the benefits that derive from their use – thus balancing access to these resources with a mechanism for sharing benefits equitably.

Access and Benefit Sharing has been incorporated in an increasing number of international regulations for genetic resources. Besides the International Treaty and the Nagoya Protocol, more recently ABS has been included in regulations on yet other types of genetic resources, such as the Pandemic Influenza Preparedness (PIP) framework and the Agreement on Marine Biological Diversity of Areas beyond National Jurisdiction (BBNJ). In each of these regulations, Access and Benefit Sharing is given substance to in a different way, ranging from multilateral (ITPGRFA, PIP, BBNJ) to bilateral (Nagoya) systems of exchange, and from systems that directly connect the provision of genetic resources to the reception of benefits (Nagoya) to systems where no direct connections are made but benefits instead are pooled in a global fund that then distributes these benefits to providers (ITPGRFA).

These different ways of institutionalizing Access and Benefit Sharing each come with distinct challenges in balancing access and fairness. The International Treaty, for example, has been relatively successful in enabling access while benefit-sharing has remained below expectations (ITPGRFA & FAO 2023a). Between the start of the system in 2007 and 2022, a mere 300.000 USD of user-based income has been collected in the global fund, in addition to 34 million USD via voluntary contributions, which includes donations by international parties who did not use the genetic resources (FAO 2023b). The bilateral system of Nagoya in turn offers relatively more certainty that benefits are shared whenever genetic resources are accessed, but Nagoya has faced more significant challenges in enabling access. For example in 2020, only 1211 exchanges were registered under Nagoya (CBD 2020), compared to 76.000 exchanges under the ITPGRFA, despite the ITPGRFA only covering a fraction of plant genetic resources. This in turn also affects the amount of benefits that are shared, since these are directly coupled to the provision of access.

Given these challenges, this paper specifically focuses on the challenges to making ABS work under the Nagoya Protocol. In the recent past there have been several challenges to this bilateral system, yet proposals to turn Nagoya into a multilateral system have failed and it seems clear that Nagoya will remain in place. Also the recent launch of the so-called Cali Fund for digital sequence information (CBD 2025) does not affect the bilateral system of Nagoya. While the Cali Fund will also cover plants that are included in Nagoya, it has simultaneously become clear that this new multilateral system will only concern digital sequence information, and that the exchange of physical genetic resources will remain embedded in the bilateral Nagoya system. In short, despite of the various challenges that have been encountered in making Access and Benefit Sharing work under Nagoya, this bilateral ABS system is here to stay. It hence remains urgent and important to identify ways in which the balance between access and fairness can be struck in more productive ways under the Nagoya Protocol.

The academic literature predominantly attributes the failure to facilitate access to challenges in implementing the Nagoya Protocol at the national level. Several authors have for example pointed out that national institutions lack commitment to provide access, and that financial, technical and human capital resources are lacking to implement the protocol in a sufficient way (Morgera et al. 2014; Robinson 2014; Halewood et al. 2021).

One hurdle that has received only scant attention thus far is the difficulty in establishing ABS agreements – the agreements where the parties involved in the exchange describe the conditions for the parties to access those resources, including how the benefits that are derived from that access will be shared. Establishing such an ABS agreement has been found to be a major stumbling block for exchanging genetic material (Heinrich et al. 2020; Ruiz Muller 2018; Schroeder et al. 2020). This is partly due to diverging expectations about the size of the benefits that should be shared, and due to a lack of clarify about the nature of the benefits that can be shared in the first place (Heinrich et al. 2020; Schroeder et al. 2020). This question over what constitutes a ‘benefit’ has only received scant attention thus far. The Nagoya Protocol itself includes a list of possible benefits that can be shared, such as up-front payments or milestone payments (CBD 2011a). However, these benefits are quite abstract and reportedly has failed to provide sufficient clarify over what benefits can be included in ABS agreements (Heinrich et al. 2020; Schroeder et al. 2020). In short, what constitutes benefit that can be shared remains poorly developed (Wynberg and Hauck 2014).

In this study, we aim to fill this gap by studying ABS agreements that have been successfully established under the Nagoya Protocol and investigating what types benefit these parties have managed to agree upon. We therefore ask what benefits have been agreed upon in successful ABS agreements under the Nagoya Protocol? We focus on agreements where plant genetic resources were shared by countries in the Global South as the objective ‘fair and equitable benefit-sharing’ of Nagoya is mainly designed to reduce inequalities for these providers (Deplazes-Zemp et al. 2018).

As we will outline below, we found that ABS agreements under Nagoya can include an incredible variety of different types of benefits. The benefits can take both monetary and non-monetary forms, they can contribute to a wide range of different objectives, and the benefits that are shared are not necessarily related to the benefits that are obtained from using the genetic resource. These insights into what benefits have been successfully agreed upon in the past, can help to tackle a main bottleneck in the establishment of ABS agreements under Nagoya by offering concrete examples of best practices. In addition, this offers an empirically-grounded and conceptually richer foundation for conceptualizing Access and Benefit Sharing under various international regulations.

The need to balance access to plant genetic resources with a fair distribution of the benefits is a challenge that is typical for what has become known as just transitions –transitioning towards more sustainable societies in way that is fair and equitable. We suggest that that the experiences in developing Access and Benefit Sharing (ABS) into a workable concept highlights the important role that regulations can play in realizing just transitions. The experiences with the concept of benefit-sharing for plant genetic resources may offer valuable insights.

The need to balance access to plant genetic resources with a fair distribution of the benefits, is a challenge that is typical for what has become known as just transitions – the aim to transition towards more sustainable societies in way that is fair and equitable. We argue that regulations on plant genetic resources and the concept of Access and Benefit Sharing (ABS) highlights the important role that regulations can play in realizing just transitions. In this context, the experiences developing Access and Benefit Sharing into a workable concept may offer valuable insights.

## Access and benefit sharing

### Understanding access and benefit sharing

Access and Benefit Sharing under the Nagoya Protocol can be understood as part of a wider shift in the conceptualization of genetic resources from a public good, available to everyone without restriction, to genetic resources as a commons. A commons refers to “a shared resource, co-governed by a community of users according to their rules and norms” (Halewood 2013).

Several rules and norms have emerged at the international level. Over the last few decades, different institutions have emerged that govern genetic resources as a commons. This includes the International Treaty for Plant Genetic Resources (ITPGRFA) that focuses on genetic resources for a selected number of staple crops that are deemed crucial for food security and agriculture; the Pandemic Influenza preparedness (PIP) framework that focuses on the exchange of human genetic resources; and the Nagoya Protocol which focuses on the exchange of genetic resources that are not included in the ITPGRFA but that still require conservation (Schroeder 2020).

Besides literature that explores the legal foundation and implementation of ABS (e.g. Girard et al. 2022; Sirakaya 2019; Ljungqvist et al. 2025), the academic literature on Nagoya predominantly focuses on two topics. First, there is a growing body of literature that explores the implications of digital sequence information (DSI). Due to technological developments at the intersection of genetics and data science, it has become increasingly easy to digitize genetic data, because of which access to the material genetic resource is no longer always necessary. This discussion revolves around the question whether the Nagoya Protocol also applies to such digitized genetic resources (e.g. Smyth et al. 2020; Aubry 2019; Aubry et al. 2021; Brink and Van Hintum 2021). This underlines the importance of understanding the relation between commons and technological innovation (Beumer et al. 2020).

Secondly, various authors focus on difficulties in implementing the Nagoya Protocol. This literature emerged in response to the widely perceived failure of the Nagoya Protocol to facilitate the sharing of genetic resources and authors have identified several factors that make it so difficult for users and providers to reach agreements of how genetic resources could be shared. Morgera et al. (2014) for example found difficulties with the engagement and commitment of the relevant national institutions that have to facilitate access to genetic resources. Coolsaet and Pitseys (2015) found that political instability caused uncertainty about the reliability of these institutions, and various other authors have pointed out there is a lack of financial, technological and human capacity to effectively implement the Nagoya Protocol (Halewood et al. 2021; Robinson 2014). Each of these factors pose a barrier to successfully achieve ABS agreements.

One bottleneck that has not been addressed is the lack of clarity about *the types of benefits* that can be shared. Perhaps this difficulty should not come as a surprise, given the fact that hardly any limits are put on the types of benefits that can be shared: the Nagoya Protocol only mentions that “benefits may include monetary and non-monetary benefits” (CBD 2011a, p. 6). As such, it is entirely open to users and providers to come up with benefits that they deem fit, and these benefits have to be concocted and negotiated separately for each new ABS agreement. The Nagoya Protocol includes a list of possible benefits that can be shared, such as up-front payments or milestone payments (CBD 2011a) but these are rather abstract and reportedly have failed to provide sufficient clarify over what benefits can be included in ABS agreements (Heinrich et al. 2020; Schroeder et al. 2020). While several studies have given advice on the negotiation process and developed model contractual clauses (CBD 2018; Schroeder et al. 2020), such standards are lacking for benefits that can be shared.

More recently, Sirakaya (2019) provided a more detailed list of benefits which she collected from frameworks that different countries developed for implementing the Nagoya Protocol. This is insightful as the conditions that are included in these frameworks shape the types of benefits that are included. However, this study is only based on a limited set of countries, including countries that have not yet ratified the Nagoya Protocol. Furthermore, a number of countries included (e.g. Uganda, Philippines, Costa Rica, Ecuador) have not shared any successful ABS agreements with the ABS Clearing House – the global platform for information exchange on Access and Benefit Sharing, and it thus remains unclear whether the benefits these countries formulated have ever been successfully included in ABS agreements. It hence remains unclear what benefits can successfully be shared in practice.

### Theoretical framework

We aim to tackle this bottleneck by identifying what types of benefits have been included in successful ABS agreements. We take a constructivist perspective to benefits in that we do not limit the benefits we include in our analysis based on a predetermined definition of benefits. Instead, anything that is included in ABS agreements as a benefit will be included in our study. This is largely unrestricted by the Nagoya Protocol as the protocol does not include binding guidelines as to what benefits can be included.

To further understand the variety and the nature of the benefits, we draw upon the work by Bram de Jonge and Niels Louwaars. In their 2009 and 2011 articles, they identified six different approaches to Access and Benefit Sharing based on the “central motivations for benefit-sharing that can be extracted from the debate with respect to plant genetic resources” (de Jonge and Louwaars 2009, p. 3). The approaches are explained in Table 1.

**Table 1:** Approaches to benefit-sharing (De Jonge and Louwaars 2009, 2011)

1. North-South imbalance: parties engage in ABS because it can promote equity international relations. This counters the situation where countries in the global North accrue economic benefits from genetic resources from the global South (Kloppenburg 2005). This approach includes benefits that redress this imbalance.	
2. **Biodiversity conservation**: parties engage in ABS because this provides resources to enable biodiversity conservation, often with the aim to conserve the diversity of genetic resources that face threats such as pollution, climate change, and human exploitation. This approach includes benefits that promote biodiversity conservation.	
3. **Biopiracy and intellectual property rights imbalance**: parties engage in ABS because it promotes equity in legal rights of plant genetic resources and related knowledge. This counters the situation where Intellectual Property Rights are used to appropriate genetic resources without compensating the local communities. This approach includes benefits that counter biopiracy.	
4. **Food security**: parties engage in ABS for a shared interest in food security. The conservation and exchange of plant genetic resources is considered crucial for food security, and benefits can be shared that strengthen that shared interest. This approach includes benefits that promote food security.	
5. **Intellectual property rights protection and public interest imbalance**: parties engage in ABS to distribute the benefits of research and development more equitably. This counters the situation where Intellectual Property Rights do not benefit all groups, for example because the appropriated genetic resources are not used as much for the needs of the global South, or because it an ‘anti-commons trap’ occurs where too many entities have exclusive rights over a resource, leading to underuse (Heller 1998). This approach includes benefits that distribute benefits of research and development more equitably.	
6. **Cultural identity protection**: parties engage in ABS to preserve and restore traditional communities and their cultures and alternative worldviews. These traditional communities may require protection in a globalizing world. This approach includes benefits that protect cultural identities of traditional communities.	

The six approaches were originally identified to understand the highly diverging interpretations of Access and Benefit Sharing and to understand the different considerations that went into designing regulations. For example, in the Nagoya Protocol, the motivation to restore the imbalance between the global North and South (approach 1) was established by allocating national sovereignty over plant genetic resources while the motivation to achieve food security (approach 4) provided the incentive to set up mechanisms for accessing genetic resources. And the motivation to protect the cultural identity of traditional communities (approach 6) was for example incorporated in different conventions by recognizing customary laws in ABS regimes.

We posit that these approaches are also helpful as a framework for understanding the variety and nature of the benefits that are shared. The different motivations for engaging in Access and Benefit Sharing each come with different types of benefits, as we articulated in Table 1. For example, if one approaches ABS as a means to safeguard biodiversity, then the benefits included in the ABS agreement should be relevant for safeguarding biodiversity, for example by funding gene banks or training farmers to conserve landraces. Yet if one approaches ABS as a means to reduce income inequality, the benefit should relate to that objective, for example by giving financial compensation to the poorest communities.

By thus categorizing the benefits in ABS agreements along the lines of the different approaches to Access and Benefit Sharing, the approaches can help to understand the variety and nature of objectives that different benefits serve.

## Case studies and methodology

### Case study selection and data collection

We focus on benefits that are included in successful ABS agreements. This limits our data to benefits that users and providers managed to agree upon, thus excluding other types of benefits that parties did not manage to agree upon, which could have for example been identified by observing ABS negotiations in real-time. The benefit of focusing on successful ABS agreements is that the benefits identified have proven to be successfully and because it allows us to include a much larger and more comprehensive sample than would be possible by studying ABS negotiations in real-time.

We collected successful ABS agreements from the ABS Clearing House database. The ABS Clearing House is a global platform for exchanging information on access and benefit-sharing under the Nagoya Protocol. Its database includes all ABS agreements that are signed by both users and providers (ABSCH n.d.). We systematically searched the database for ABS agreements for plant genetic resources in developing countries, resulting in a comprehensive list of all successful ABS agreements. We found a total of 102 ABS agreements in nine different countries (see Table 2).

**Table 2:** ABS agreements per country

Country	ABS agreements
South Africa	29
India^1^	23
Kenya	14
Peru	14
Panama	13
Mexico	7
Benin	3
Guatemala	2
Ethiopia	1
Total	103

^1^India divides their IRCCs in four distinct categories based on different sorts of applications. In our sample, we included ABS agreements under Form I, which covers agreements on access to biological resources and/or associated traditional knowledge. Agreements under form III covers IPR and was excluded. Form II covers agreements on transferring the results to foreign companies and Form IV covers agreements for transferring samples that already have been accessed to third parties. India has a relatively standardized approach that strongly encourages monetary benefits. The benefits identified in cases from Form I are therefore representative for all cases in India, as was confirmed by the interviewees

The ABS agreements include relatively short and concise descriptions of the benefits that are shared. In order to gain a more in-depth understanding of these benefits, we complemented the agreements with both grey literature and semi-structured qualitative interviews. These enable us to gain a deeper understanding of the benefits that are shared, which is crucial for answering the research question.

Because it was not feasible to do so for all 102 agreements, we focused on a smaller selection. In making this selection, it was important to include ABS agreements in a diverse set of countries. Countries can make their own rules and procedures for accessing and sharing of benefits (Brink and van Hintum 2020; CBD 2011a) and this means that different types of benefits could occur in the different countries. We therefore aimed at including at least two agreements for each country (except for Ethiopia that only had one agreement). In addition, we aimed to interview both users who gained access to genetic resources (consisting of diverse actors such as companies, botanical gardens, and research institutes), providers of the resource who facilitate access to the genetic resource (including actors such as the competent national authority or civil society organizations), and the National Focal Points involved in each of the agreements.

**Fig. 1:**
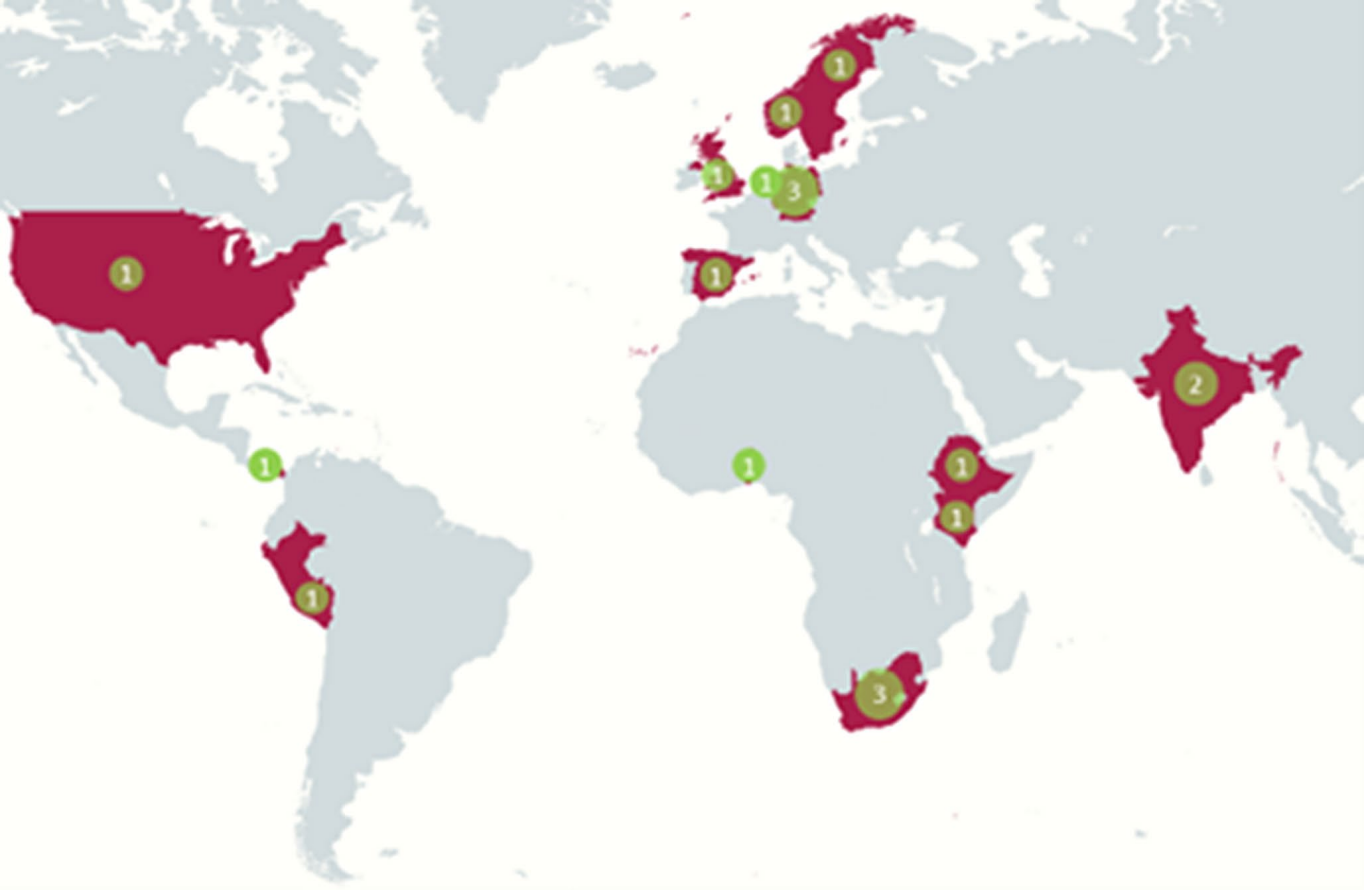
Geographical location of interviewees

We contacted 65 different users, providers, and focal points and eventually conducted 18 interviews. An overview of the geographical location of the interviewees can be found in Fig. 1. Interviews lasted between 45 and 80 min and were held via Microsoft Teams. One interview was held with a Spanish interpreter. The interview guide included both general questions about the concept of Access and Benefit Sharing and lessons learned, and questions covering the different motivations in the framework of De Jonge and Louwaars. In total, the interviews covered 16 different agreements for gaining access to genetic resource in ten different countries – three from India and South Africa, two from Mexico and Panama, and one each from Ethiopia, Benin, Guatemala, Kenya, and Peru. For the grey literature search we used both Google and Google Scholar. The search terms used can be found in appendix A.

After collecting the data from the agreements, interviews, and written documents, we coded and analyzed the data in Microsoft Excel. We first coded each agreement for the plant type, country of origin, actor type, objective of access, and involvement of local actors. We then developed codes that allowed us to group individual benefits in specific agreements. This allowed us to identify types of benefits that occurred in different agreements. Each benefit was furthermore categorized using the framework of De Jonge and Louwaars. For example a benefit like “funding research on biodiversity conservation in Peru” was coded under “biodiversity conservation”. Once the process of coding was finished, we rationalized the codes by re-reading all the codes and coded interviews, and eliminating all double codes (Bryman 2012). Finally, we analyzed the findings by looking for patterns in the data, for example looking for correlations between the types of benefits that are included with the types of actors involved in the negotiation, the geographical location, the date of the agreement, etc.

**Table 3:** Case studies of successful ABS agreements

Agreement	Plant species	Access objective	Provider country	User country
AC1	Osiris Species	Commercial	Ethiopia	Ethiopia/India
AC2	Baobab	Commercial	Kenya	Kenya/Germany
AC3	Herbal plant	Commercial	South Africa	South Africa
AC4	Herbal plant - Mexican arnica	Commercial	Mexico	Spain
AC5	Resurrection bush	Commercial	South Africa	South Africa
AC6	7 different species	Commercial	India	India
AC7	Maize	Commercial	Mexico	USA
AC8	Rooibos plant	Commercial	South Africa	
AN1	Symbiose plant and ants	Non-commercial	Peru	Germany
AN2	Fungi	Non-commercial	Benin	Germany
AN3	Discovery of new plant species Himalaya	Non-commercial	India	United Kingdom
AN4	Discovery of new plant species	Non-commercial	Indonesia	United Kingdom
AN5	Symbiose Chelymorpha and host plant	Non-commercial	Panama	Panama
AN6	Seagrass	Non-commercial	Panama	Germany
AN7	TK on dioecious species	Non-commercial	India	Norway
AN8	Mahogany	Non-commercial	Guatemala	Worldwide
ACN1*	*No specific plant*	Non-commercial/ commercial	Peru	
ACN2*	*No specific plant*	Non-commercial/ Commercial	Panama	Germany
ACN3*	*No specific plant*	Non-commercial/ Commercial	India	Worldwide

AC = Agreement Commercial; AN = Agreement Non-Commercial; ACN = Agreement Commercial / Non-Commercial; *did not talk about a specific ABS agreement

## Results

### Main results

Our main finding is that a wide variety of different benefits are included in ABS agreements. In the sixteen agreements, we found nineteen different types of benefits (see Table 4). This includes both monetary and non-monetary benefits, ranging from paying a percentage of the sales to national authorities to offering employment opportunities, and from purchasing plants for a fair price to co-publishing results and or teaching local communities about biodiversity. In addition, we also found a range of other benefits that were shared but that were not included in the formal agreements. We will call these ‘external benefits’. In other words, we found ABS agreements can include an incredibly diverse set of benefits.

These benefits could be shared under almost each of the different approaches to Access and Benefit Sharing. The benefits contributed to address the imbalance between the Global North and Global South; conserve biological diversity; tackle biopiracy and the imbalance in property rights; share interest in food security; address the imbalance between IPR protection and the public interest; and protect the cultural identity of traditional communities. This once more underlines the diverse nature of the benefits we identified.

In the next sections we will describe the monetary (“Monetary benefits”), non-monetary (“Non-monetary benefits”), and external benefits (“External benefits”) and explain what approaches of Access and Benefit Sharing they contribute to. In Sect. “Analysis” we will then analyze these results in more depth.

**Table 4:** Overview of types of benefits

Number of times included	Total	CU*	NCU*	Objective
**Monetary benefits**				
• Sales percentage	4	4	-	North-South imbalance (4)
• Profit percentage	1	1	-	Biopiracy and IPR imbalance (2)
• Plant cultivation purchasing	9	9	-	North-South imbalance (5), biodiversity conservation (3), food security (1)
• Upfront payment	1	1	-	North-South imbalance (1)
• Annual license fee (royalty percentage)	1	1	-	North-South imbalance (1)
**Non-monetary benefits**				
• Sharing of research results	9	3	6	North-South imbalance (6), food security (1), IPR-public interest imbalance (1), cultural identity protection (1)
• Employment opportunities of local communities	1	-	-	North-South imbalance (1)
• Sharing research equipment	2	-	2	North-South imbalance (1)
• Training the students of country of origin	3	-	3	North-South imbalance (2)
• Sharing authorship	5	-	5	North-South imbalance (6), IPR-public interest imbalance (6)
• Training local communities on cultivation	3	3	-	Biodiversity conservation (3)
• Helping socio-economic projects of local communities on conservation efforts	1	1	-	Biodiversity conservation (1)
• Participatory research: incorporation of local communities in research	3	1	2	Biodiversity conservation (1), Cultural identity protection (1), North-South imbalance (1)
External benefits				
• Financing/helping socio-economic projects of local communities on basic needs	3	3	-	North-South imbalance (4)
• Giving small incentive to local communities for attending research	2	-	2	North-South imbalance (2)
• Trust funds for conservation efforts	1	1	-	Biodiversity conservation (1)
• Teaching local communities about biodiversity	3	-	3	Biodiversity conservation (3)
• Knowledge generation about national biodiversity	7	-	7	Biodiversity conservation (7)
• Sharing authorship	1	-	1	North-South imbalance (6), IPR-public interest imbalance (6)
**Total**	60	28	32	

* CU = commercial use, NCU = non-commercial use

### Monetary benefits

We found five types of monetary benefits: paying a percentage of the sales of the end-product, purchasing the plant from local communities, paying for labor by community members in cultivating the plant, an upfront payment, and an annual license fee. With the exception of one ABS agreement, these monetary benefits are all part of commercial use agreements.

In most cases the amount of moeny is relative to the value produced by using the genetic resource. Four agreements for example include the benefit to pay a certain percentage of the sales of the end-product that will be developed using the plant genetic resource in question. The percentage lies between one and three% and will be paid to the national authorities. In another agreement, the provider country will receive a percentage of the net profits (instead of sales) of the end-product. In this way, the more the end-product is sold, the more benefits will be shared.

This is also the case for agreements where users agree to purchase the plants containing the genetic resource from local communities in the provider country. For example in the rooibos agreement in South Africa, the user agreed to purchase the rooibos from South African farmers and to pay an additional 1,5% on the farm gate price. And the arnica agreement in Mexico includes the benefit that the company using the genetic resources will purchase the plant in question exclusively from the local community. This means that the more products using the genetic resource are sold, the higher the benefits that will be shared. Agreeing to purchase the plant from farmers in the provider country ensures a steady supply to the user while providing additional benefits to the provider of the genetic resource.

In one case, the benefits are not relative to sales or profits over a particular end-product but to the income derived from intellectual property rights. This is the case for a maize variety in Mexico that contains a self-fertilizing gene that helps the plant fix its own nitrogen (see Pskowski 2019). The company is allowed to acquire intellectual property rights over this genetic resource and in return the company will share 50% of any patent royalties with the community.

We further found two types of monetary benefits that are not tied to the end-product. The agreement on the use of the Osiris plant in Ethiopia includes an upfront payment of 50.000 US dollar to the national authorities, and the same agreement also includes an annual license fee of 2000 dollars. For these benefits, the amount of money shared remains the same regardless of how much value is created with the genetic resource.

The monetary benefits contribute to about half of the approaches to Access and Benefit Sharing identified by De Jonge and Louwaars (2009; 2011). In the majority of the cases, the monetary benefits redressed the imbalance between the global North and global South. The agreements to share a portion of the value created by the genetic resource are a prime example. If users in the global North would keep all the value they generate by using the genetic resource, then inequality between the North and the South would increase. Giving a share of these benefits to the providers of the resource reduces that inequality. The same is true for upfront payments, annual license fees, and purchasing the plant from local communities, who thereby profit from providing access to the genetic resource. These measures may not reduce inequality between the global North and the South proper – after all, it may well be that users in the North proportionally obtain more benefits from the genetic resource than they share with the providers in the South, thus effectively increasing inequality. Yet these measures do reduce the North-South imbalance by ensuring that the global South also derives benefits from the genetic resources in a way that can certainly be considered more fair than if these benefit-sharing arrangements were absent.

We did not find any monetary benefits that contribute to food security, addressing the imbalance between IPR and public interest, or protecting cultural identities. However, we did find monetary benefits that contribute to biodiversity conservation and fighting biopiracy. The latter is for example the case in the agreement on the self-compatible Mexican maize variety where the company shares 50% of patent royalties. This overcomes biopiracy by including compensation to the local community in private appropriation of the genetic resources.

Monetary benefits also contribute to biodiversity conservation. For example the agreement to purchase plant from local farmers is not only deemed beneficial for generating income among local farmers, it is also meant to benefit biodiversity conservation. By offering income certainty, this monetary benefit “incentivizes the local communities to do to conserve and sustainably utilize their resources” (interview AC1). And in the case of a Mahogany tree in Guatemala, the public research institute that gained access over the genetic resource temporarily pays community members for helping out with the fieldwork. These payments are meant to acknowledge and legitimize community members’ engagement and responsibility for their surroundings, in the hope that after the research and the payments have ended, the community members continue to breed their own Mahogany plants, thus contributing to biodiversity conservation.

### Non-monetary benefits

We also found a wide range of different non-monetary benefits: nine in total. These are shared by both commercial and non-commercial users.

The non-monetary benefits that were found most often are related to research. The most common was to share authorship with research institutes in the providing country on scientific publications resulting from the access to the genetic resource. This was part of almost all agreements, both for commercial and non-commercial use. Sharing authorship is not merely cosmetic, as it often implies that the research should be conducted together. One interviewee from the provider country noted that it is important to make this explicit: “Not that the other researcher [accessing the genetic resource] will write the paper alone and, in the end, write my name on this. That is cheating and not normal. There should be an equal contribution in the paper” (AN2).

Another non-monetary benefit that was regularly shared was to share the results from the research about the plant genetic resource. For example the company that sought to access genetic resources of the Arnica plant in Mexico, agrees to share the results of this research with a Mexican university. Sharing research results is part of almost all agreements and this is also suggested by the Convention on Biological Diversity (CBD 2011a). Nevertheless, four interviewees critically observe that whereas this may benefit researchers in the provider country, local communities do not always benefit from this. The research shared is not always of practical use and academic results are often difficult to interpret for local communities.

Other research-related benefits include the sharing of research equipment that otherwise cannot be afforded by the provider country (which occurs in two non-commercial agreements), and training students from the provider country, for example by funding scholarships, summers schools, on-the-job training and giving courses locally. In the case of the Mahogany plant in Guatemala, the ABS agreement includes the benefit that local community members (as opposed to researchers) will participate in the research. Such ‘participatory research’, as it is dubbed, increases the likelihood that the findings will benefit the local communities (Van Zonneveld et al. 2018). An interviewee explained that local community members “were really involved in the research and it was also clear that the results of the research could also be used by themselves” (AN8).

While the research-related benefits are included most frequently, we also found a range of other non-monetary benefits. One agreement for example includes employment opportunities for local communities. This is included in the ABS agreement on an Osiris species in Ethiopia, where the user of the genetic resource agrees to build a factory in Ethiopia and to hire employees from the vicinity. As a result, one interviewee stated, an estimated “125 Ethiopians are provided with permanent employment” (AC1). One agreement further offers various types of non-monetary support to socio-economic projects of local communities and three contracts also include the agreement to train local communities in cultivating the crop in question, which in some cases was combined with the agreement that the user will purchase the crop from the communities. For example the company accessing the Arnica plant in Mexico helps installing irrigation for watering the plants, that are cultivated in a relatively dry area.

Except for biopiracy, the non-monetary benefits contribute to each of the different motivations for benefit-sharing that were identified by De Jonge and Louwaars (2009, 2011). The majority address the imbalance between North and South. For example, according to the interviewees, benefits such as co-authorships can reduce the imbalance between North and South by enabling universities in the South to climb university rankings and increasing the chances of local researchers for competitive research grants.

But we also found non-monetary benefits that contribute to other motivations. The training that is provided to local communities on cultivating plants, for example, contributes to biodiversity conservation. The prime example here is the training on Osiris cultivation in Ethiopia. In this case, the plant population had been declining due to overexploitation. The company “supplied a training manual (…) which gives step-by step instructions on how to grow Osiris from seed, air layering and cuttings. The manual was translated into local languages” (AC1). The regions where this manual was provided later had a steadier Osiris population, thus supporting the conservation of Osiris.

In several cases, a single benefit serves multiple objectives. This is for example the case for several research-related benefits. According to our interviewees, sharing research results can both help reduce the imbalance between the North and South by helping Southern researchers to stay up-to-date and increasing the engagement of the global South in international research (Martins 2020).^2^ While also serving other objectives. For example the agreement on Baobab seeks to share research results with the aim to increase food security. The knowledge that is shared helps researchers but also helps local communities to obtain additional nutritional value from the plant.

In one particular case, sharing research results contributed both to protect cultural identity, fight biopiracy, and address the imbalance between IPR and public interest. This concerns the case where research is shared on traditional knowledge on dioecious species of Indian folk healers. Sharing the research results helps to store traditional knowledge that is under threat. As one interviewee noted, “people are forgetting the traditional knowledge. You think the younger generation is not following the tradition. They are moving out of their village. They do not want to live in the forest with their parents. So, the traditional knowledge is getting lost and documenting this knowledge is a benefit for Indians” (AN7). At the same time, sharing this knowledge prevents it from being patented without the inclusion of the traditional knowledge holders, thus fighting biopiracy. And by stipulating that the knowledge about the genetic resource could only be used for non-commercial purposes, the agreement also addresses the imbalance between IPR and the public interest. If the researchers want to use the findings for commercial use, a new ABS agreement has to be negotiated.

In short, we found a variety of non-monetary benefits, that contribute to all the different objectives for benefit-sharing from De Jonge and Louwaars (2009; 2011), with some benefits even contributing to different objectives at once.

### External benefits

We also found various benefits that were not included in the formal agreements, yet that users and providers nevertheless agreed upon in the process of formalizing ABS agreements. We will call these ‘external benefits’ as these were ‘external’ to the formal agreement. We include these in our analysis because these benefits were part of the overall negotiation on benefit-sharing, despite not being part of the formal ABS agreement. Moreover, the fact that we found examples of such benefits not just on one or two cases but in almost all agreements, indicates that external benefits may be a common elements of Access and Benefit Sharing.

The nature of the external benefits is as diverse as the benefits described in the previous sections. External benefits are part of both commercial- and non-commercial use cases and include both monetary and non-monetary benefits.

In six cases, the external benefits contribute to restoring the imbalance between global North and South (Table 3). This was for example true for four commercial cases where users fund and support socio-economic projects in local communities. This concerns different types of projects, including the construction of a school and a creche, educating teachers, and scholarships for students. In the case of arnica in Mexico, the cosmetics company help women to start their own micro-enterprise selling cosmetics. They finance courses on cosmetics and cosmetics production and they finance the facilities to produce new cosmetics (also see UNDP 2018). In one case, the company in question has a relatively modest turnover and argues they cannot invest in the type of larger socio-economic projects that they deem most effective: “when it comes to benefit sharing, we do not have millions of millions to share, it is relatively modest amounts” (AC3). They therefore will start a foundation that will actively seek additional funds from their partners in Europe, so as to collect reach a larger amount of money to invest in socio-economic projects.

In eleven cases, external benefits contribute to biodiversity conservation. This includes sharing knowledge generated about national biodiversity (also if the purpose of accessing the genetic resource was different). This is part of almost all non-commercial use cases. In other cases also local communities are taught about biodiversity. For example, in the case of the symbiose plant in Peru, the researcher accessing the genetic resource trains local tour guides about local biodiversity.

The interviewees mentioned various reasons why these external benefits are not included in the official agreements. In most cases, it was too difficult to formulate these benefits in sufficiently concrete terms. For example in some commercial-use cases, companies argued that it was too hard to quantify the benefits the company could offer because there was too much uncertainty over the commercialization of the end-product. This difficulty is also encountered in other regulations (e.g. Beumer 2019) and is especially challenging when standards are absent.

In some cases, the decision to exclude these benefits from the formal agreements may indeed reflect the difficulty in making these benefits sufficiently concrete (or the lack of capacity to do so). That being said, however, the exclusion of these benefits from formal agreements may also be testament to a negotiation process in which users gained the upper hand. After all, benefits that are external in some cases, such as the sharing of research results and the financing of socio-economic projects, are included in formal agreements in other cases, thus showing that formalization certainly is possible. The same is true for the argument that benefits cannot be included in the agreement due to uncertainty over commercialization: other agreements show that this too could have been resolved by making the benefits shared relative to the benefits derived from the commercial use. The only difference between including and excluding these benefits in the ABS agreements, is that external benefits are not legally binding.

### Analysis

One thing that stands out from the findings is that monetary benefits are mostly included in commercial use agreements: monetary benefits are part of all eight agreements for commercial use, whereas none of the eight agreements for non-commercial use in our sample include monetary benefits.

At first sight, this may not be surprising. In non-commercial use agreements, the use of the genetic resource will not result in monetary benefits for the user, and hence no monetary benefits are shared with the provider, while the reverse is true for commercial-use agreements. However, we found that the benefits that are shared are oftentimes unrelated to the benefits that the users derive from the genetic resources. For example, in three out of eight commercial cases, companies initially planned to only share non-monetary benefits, despite the aim to derive monetary benefits from the use of the resource themselves.

In each of these cases, the non-monetary benefits were rejected by local communities or national authorities – not because the companies would derive monetary benefits from the use of the resource but for entirely different reasons. The main reason for preferring money over non-monetary benefits is that money can relatively easily be divided over different groups. This is particularly important in cases where communities are geographically and politically dispersed. As one interviewee observed, oftentimes “there is not one leader in the community that takes care of it but there are multiple people spread around the country that needs to negotiate with each other. Therefore, financial benefits that we accrued to these traditional knowledge holders are easier to administer than the projects. So, it is easier to have money” (AC3).

Also in the case of India, where commercial agreements only include monetary benefits, monetary benefits are preferred because it helps to simplify the process of establishing ABS agreements. These monetary benefits are transferred to a biodiversity management committee of the government which then decides what happens with the money, without having to negotiate that with the user of the resource. According to Sudhi (2019), the biodiversity management committee allocates most of the money to socio-economic projects. In such cases, the monetary benefits are preferred because they offer more autonomy in deciding what non-monetary benefits are most suitable.

**Fig. 2:**
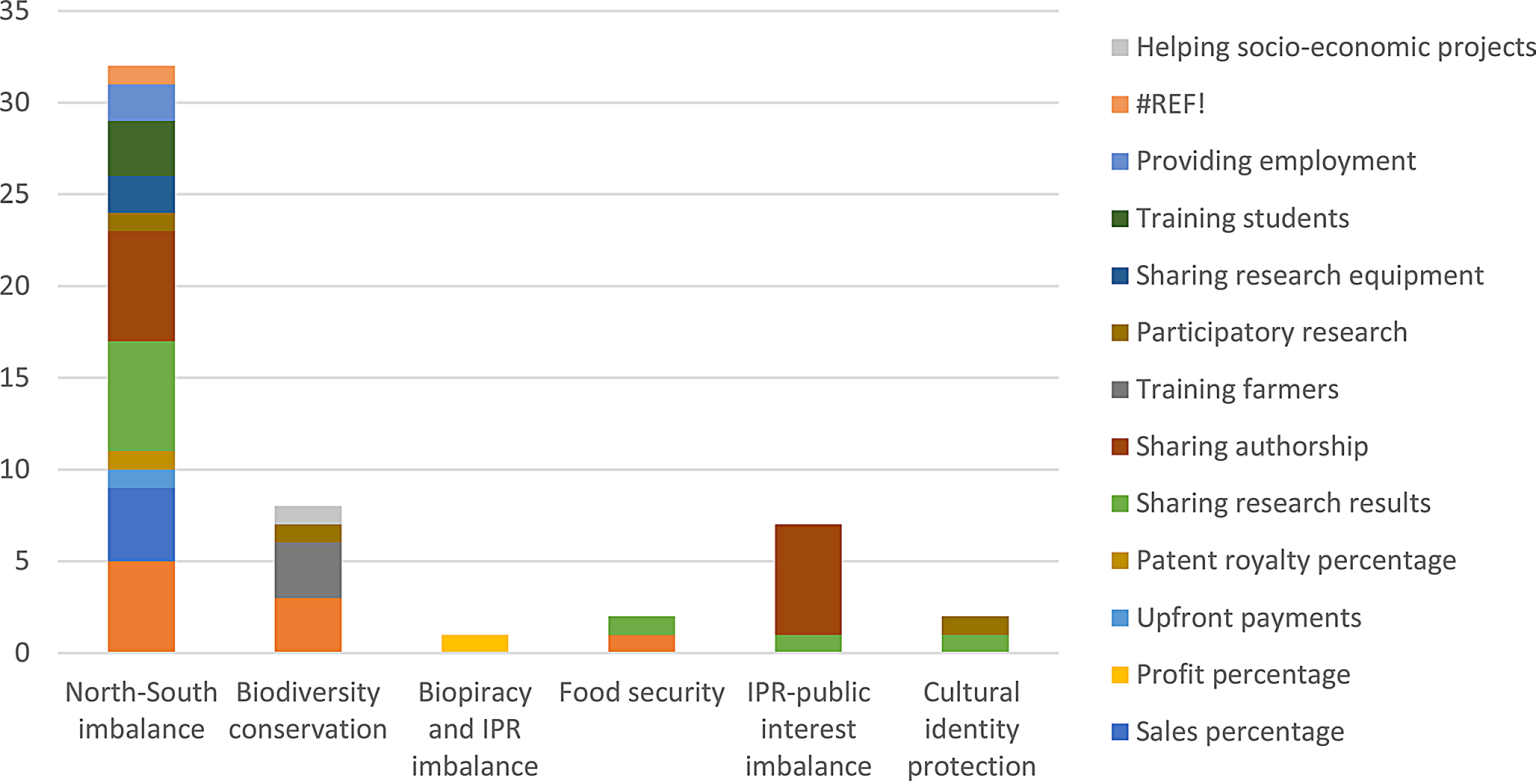
Distribution of monetary and non-monetary benefit types per ABS approach

A second finding relates to the approaches to Access and Benefit Sharing identified by De Jonge and Louwaars (2009; 2011). We found ABS agreements contribute to all the different approaches. The majority of benefits contribute to redressing the imbalance between the global North and South but we also found benefits that contribute to biodiversity conservation, fighting biopiracy, addressing food security and the imbalance between IPR and public interest, and protecting cultural identity (see Fig. 2). This once more underlines that the benefits that users share are oftentimes unrelated to the benefits that they derive from using the genetic resource.

## Conclusion and discussion

### Conclusion

Access and Benefit Sharing is an emerging concept in combining the goal of ensuring wide access with the objective of doing so in a fair way. Done successfully, it incentivizes biodiversity conservation and enables plant genetic resource to be widely used for innovations that benefit sustainability and food security, while simultaneously ensuring that the benefits that are derived from those resources are distributed fairly. In the case of the Nagoya Protocol, however, relatively few genetic resources have been exchanged since the ABS system came into effect in 2014, also compared to multilateral ABS systems. One of the factors that stands in the way of reaching ABS agreements under Nagoya is the lack of clarity as to what benefits can be included. This creates regulatory uncertainty and increases transaction costs. To this end, we identified the benefits that are shared in successful ABS agreements. We hope this contributes to removing one of the barriers facing the ABS system.

Our main finding is that a wide range of different benefits have been included in successful Access and Benefit Sharing agreements of the Nagoya Protocol. These are summarized in Table 4. This includes for monetary benefits such as percentages of the profits, upfront payments, and purchasing plants from local farmers; and it includes non-monetary benefits such as training local communities in plant cultivation, sharing research results and equipment, and projects for local socio-economic developments.

These benefits are also very different in nature, as they contribute to highly diverging objectives. We found that most benefits aim to reduce inequality between the global North and the global South, but we also found benefits aiming to counter biopiracy, conserve biodiversity, promote food security, restore the balance between intellectual property and public interests, and protect cultural identities. The diversity of these benefits is furthermore underscored by the finding that the benefits that are shared are not necessarily related to the benefits that are obtained from using the genetic resource. While this is most often the case, for example by sharing research results or a share of the profits, we also found various cases where this was not the case – including building factories to provide employment, installing irrigation facilities, and offering courses on cosmetics production.

This overview and characterization of benefits shared in successful ABS agreements contributes to reaching successful ABS agreements by providing more clarity over what benefits can be shared.

### Discussion

Based on our findings, we would like to suggest some avenues for future research. First, besides the lack of clarity as to *what* benefits can be included, ABS agreements under bilateral systems such as Nagoya are also hindered by diverging expectations regarding the magnitude of these benefits. While such differences are a normal part of negotiation and actors are free to propose benefits of any size, we believe the process could be strengthened by additional insight into what constitutes a proportionate level of benefit-sharing. Of particular help are studies that pinpoint the relative value that a specific genetic resource adds to an end product. This helps users to critically assess offers that are only a tiny proportion of the added value of the genetic resource and it helps providers to critically assess requests that greatly outnumber the value derived from the resource.

Second, another question that is still wide open is the effectiveness and impact of benefit-sharing. This is perhaps not surprising considering the different aims that benefit-sharing can serve (De Jonge and Louwaars 2009). Nevertheless, it is remarkable that there is currently very little understanding of the effectiveness of different benefit-sharing sharing arrangements. This is not only the case for the bilateral Nagoya system, but also for the multilateral form of ABS under the International Treaty. We only found a handful of studies that have begun to investigate this. Barizah and Winarsi (2020) for example specifically focus on the impact of benefit-sharing on empowering local communities. They conducted three case studies using legal resources and found that ABS agreements under Nagoya can strengthen the recognition of traditional knowledge. Ramsdell et al. (2016) in turn focus specifically on the effectiveness in achieving biodiversity conservation through Nagoya, finding that combining monetary and non-monetary benefits works best for conservation efforts, as farmers who received both money and training continued to cultivate and conserve the plant long after the financial incentive had stopped. These studies only scratch the surface regarding the effectivity of ABS and more insight into how benefit sharing impacts different groups and what benefits are most effective in achieving certain objectives, can provide valuable input for parties to negotiate what benefits should be included in ABS agreements.

The third suggestion for future research is to explore the specific conditions which make certain types of benefits suitable. Our results already give some indications for this. For example, we found that if the community is geographically or politically dispersed, monetary benefits may be most suitable, as these are relatively easy to divide. While also in this case users and providers are free to propose whatever benefits regardless of the conditions, we nevertheless think that identifying patterns and arguments for connecting specific types of benefits to specific circumstances may ease such negotiations. These suggestions for future research will help in formulating ABS agreements under Nagoya, thereby facilitating the process of providing access to genetic resources in a fair way. For multilateral systems that collect and share benefits via a central fund, such studies could also offer valuable insights into what types of benefits could best contribute to achieving biodiversity conservation in a fair manner.

The concept of Access and Benefit Sharing is a prime example of an attempt to balance the tension between access and fairness. This tension, however, is also present in many other types of regulation for food and agriculture – whether these regulations seek to promote innovation (e.g. intellectual property rights or breeders’ rights), conserve biodiversity (e.g. the Convention on Biological Diversity), enable climate adaptation measures (e.g. the Adaptation Fund under the Kyoto Protocol), or protect human and environmental health (e.g. regulations for genetically modified organisms).

We expect these tensions to become ever more important in the context of climate change. The urgent need for transformative change towards more sustainable societies inevitably comes with a host of implications for inequality and fairness – a tension that has recently gained visibility under the term ‘just transitions’. In this context, the experiences with the concept of benefit-sharing for plant genetic resources may offer valuable insights into the role that regulation may play in realizing just transitions.

## Appendix A

Search terms for grey literature search.

**Table:** 

“ABS agreement” OR “access and benefit-sharing” OR “Nagoya Protocol”	AND	Country of origin (Ethiopia, Kenya, Benin, South Africa, Mexico, Panama, Guatemala, India)	AND	“Name of specific genetic resource” (see Table 3)
			AND	Name of specific region of genetic resource
			AND	Name of user (company/research institute)

## Footnotes

2 Several interviewees critically questioned these benefits, stating that these may benefit researchers in the provider country but that local communities do not always benefit from sharing research results.
